# Supplementary material for: Adherence to DASH Dietary Pattern and Its Association with Incident Hyperuricemia Risk: A Prospective Study in Chinese Community Residents
Source: Nutrients. 2022 Nov 16;14(22):4853. doi: 10.3390/nu14224853 (PMC9692761; doi:10.3390/nu14224853)
Supplement: Supplementary file 1 [file nutrients-14-04853-s001.zip › nutrients-2046321-supplementary (1).pdf]

Table S1. Identification of incident hyperuricemia among participants who completed the first in-person survey.

Diagnosed by physical measurements in the face-to-face survey	Diagnosed in the local health information systems		
	No	Yes	Total
No	8475	289	8764
Yes	496	110	606
Total	8971	399	9370

Table S2. SUA levels (μmol/L) of participants according to sex and number of cardiometabolic diseases.

Sex	Cardiometabolic Diseases		
	0	1−2	3−4
Men	308.89	323.37	329.26
Women	244.44	268.29	290.66
Total	263.34	288.21	305.86

Abbreviations: SUA, serum uric acid.

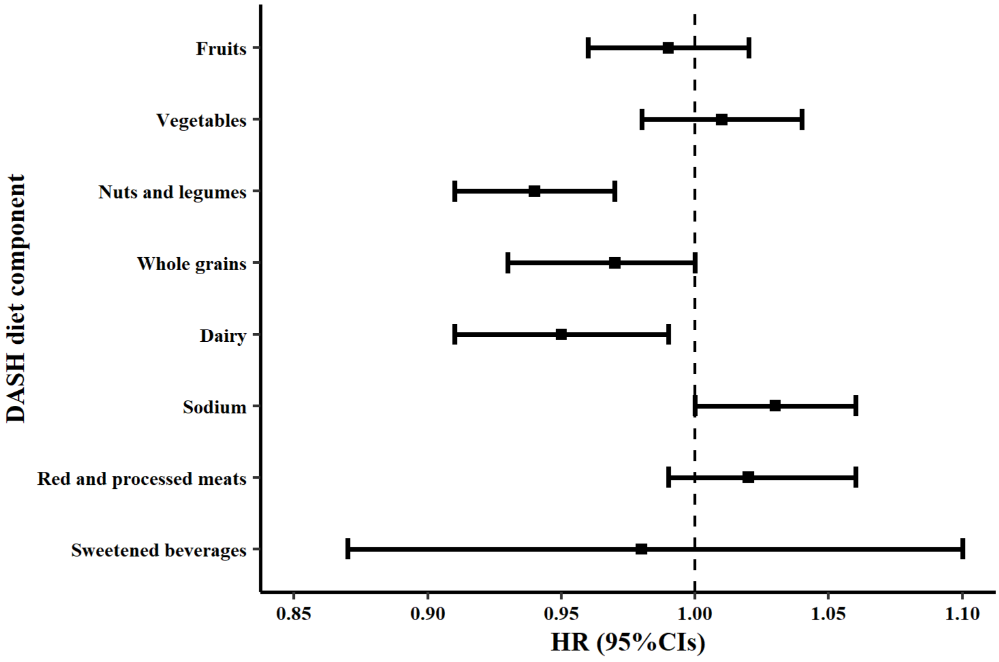

Figure S1. The Association of DASH Diet Components Scores with Incident Hyperuricemia. Fully-adjusted for age, sex, marriage status, education level, family history, BMI groups, hypertension, diabetes, hyperlipemia, smoking, alcohol drinking, PA, and total energy intake at baseline.
